# A low-cost imaging platform for quantitative characterisation of scaffold surface macrotopography with potential application in craniofacial tissue engineering

**DOI:** 10.1007/s11517-026-03661-6

**Published:** 2026-08-28

**Authors:** X. Marimon, E. Saman-Sakkal, R. Rodriguez, A. Portela, M. Cerrolaza, M. A. Mateos, R. Pérez

**Affiliations:** 1 Department of Strength of Materials and Structural Engineering, Universitat Politècnica de Catalunya (UPC-Barcelona TECH), 08028 Barcelona, Spain; 2 Institut de Recerca I Innovació en Salut (IRIS), Barcelona, Spain; 3 University College Dublin, Belfield, Dublin 4, Ireland; 4 Institut d’Investigació i Innovació Parc Taulí (I3PT), 08202 Sabadell, Spain; 5 Bioengineering Institute of Technology, Universitat Internacional de Catalunya, 08195 Sant Cugat del Vallès, Spain; 6 School of Engineering, Science and Technology, Valencian International University (VIU), 46002 Valencia, Spain

**Keywords:** Tissue engineering, Scaffolds, Pores, Craniofacial, Feature extraction, 3D printing, Image processing

## Abstract

Craniofacial bone regeneration is a demanding application of tissue engineering (TE) in which scaffold architecture—pore size, shape and spatial distribution—is a determinant of regenerative success, so that reliable geometric quality control is a prerequisite for translation. Conventional assessment of printed scaffold geometry is largely manual, time-consuming and subject to operator variability. Here we present a low-cost, semi-automated imaging platform that combines custom 3D-printed hardware—a 12.3 MP Raspberry Pi High Quality Camera with a 6 mm CS-mount lens, an adjustable monopod and a ring illuminator—with a dedicated image-processing pipeline. The system acquires a single zenithal image of a scaffold and segments it to quantify the number, area, perimeter and compactness of the pores of the uppermost printed layer; it therefore characterises two-dimensional surface macrotopography and does not resolve internal three-dimensional architecture or pore interconnectivity. On a printed reference grid the platform was highly repeatable (coefficient of variation, CV = 1.33%) but systematically underestimated pore area by 11.6%, a reproducible bias that can be removed by calibration. On a 3D-printed PLA scaffold, repeated acquisitions agreed to within CV = 0.01–2.48% (N = 5), whereas manual measurement of the same specimen by three experienced operators gave an inter-user CV of 11.5–22.8% and an intra-user CV of 0–12.7%. Feasibility was further demonstrated on two silica–gelatin hybrid bioink scaffolds and on a silica-based scaffold with non-linear pore boundaries. The total hardware cost is approximately €170. By reducing user-dependent variability under the conditions tested, and at a cost accessible to standard laboratories, the platform provides a practical quality-control tool for scaffold fabrication, with potential application to craniofacial tissue engineering.

**Graphical abstract:**

**Figure:**
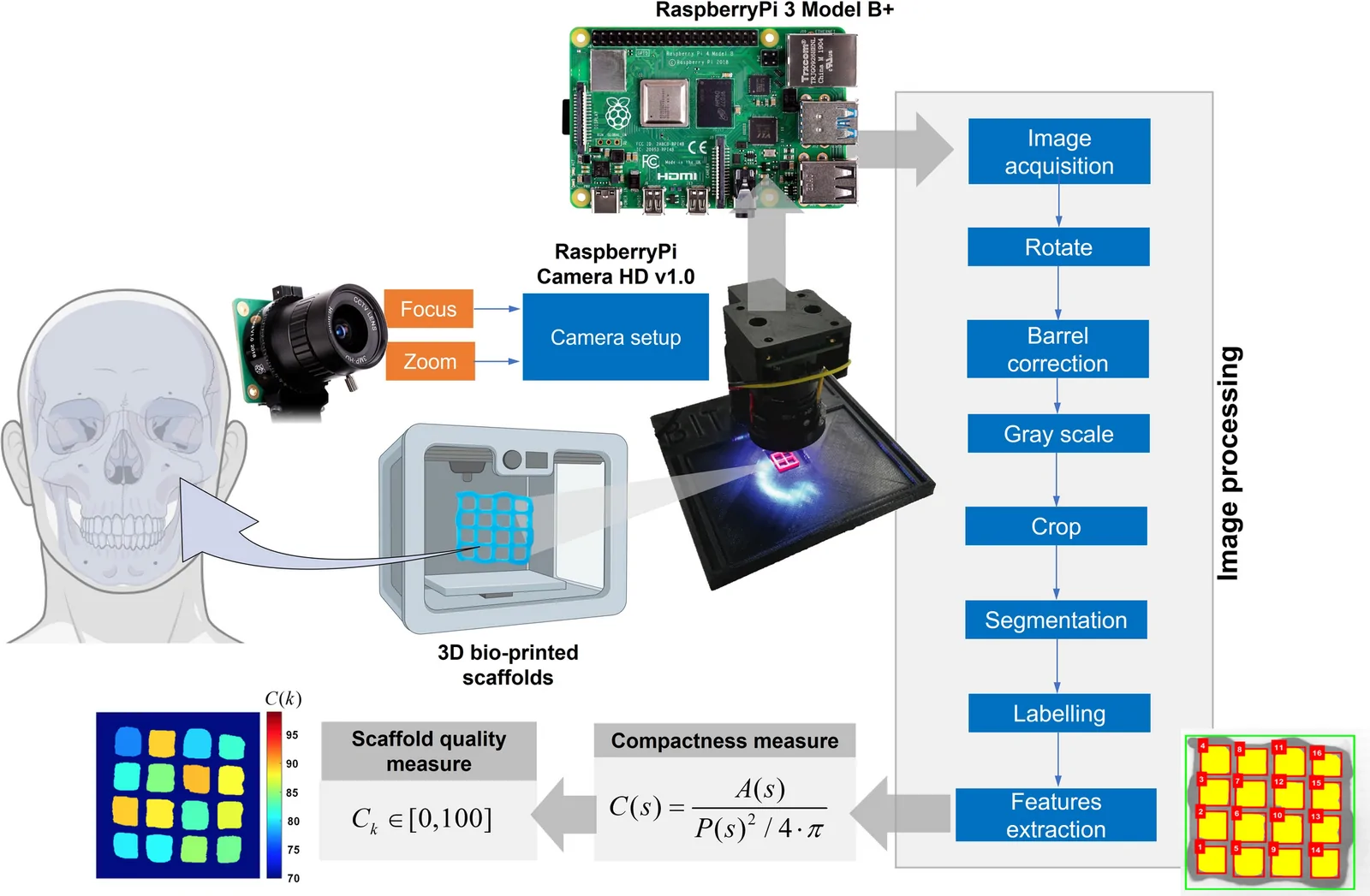

## Introduction

One of the three key elements of classical tissue engineering (TE) is the scaffold. Scaffolds provide structural support for cell attachment and subsequent regeneration (O’Brien [1], Chan and Leong [2]). In the context of craniofacial regeneration, where the restoration of complex bone and soft tissue structures demands precise scaffold architectures, the semi-automated platform presented here is intended to support reliable assessment of scaffold shape fidelity and surface macrotopography. This capability can accelerate the development of patient-specific scaffolds that better replicate native tissue environments, thereby improving regenerative outcomes as reported by Germaini et al. [3] and Turnbull et al. [4], among others. Thus, a scaffold designed for craniofacial regeneration is primarily defined by its mechanical and geometrical properties, which play a crucial role in guiding tissue development and structural organization (Engler et al. [5], Quan et al. [6]).

The size and shape of pores—the void spaces within a scaffold—are two characterising parameters that influence the functionality of the construct. Traditional methods for manufacturing scaffolds include particle-leaching casting, foaming, phase separation, melt moulding, and electrospinning (Carletti et al. [7]). Although these techniques allow the relatively simple fabrication of porous scaffolds, they lack precise control over the external geometry of the scaffold and its internal porosity (i.e., pore size, shape, distribution, and interconnectivity), which significantly impacts the effectiveness of the regenerative process (Bružauskaitė et al. [8], Bidan et al. [9]).

The adoption of Additive Manufacturing (AM) techniques in TE has revolutionised the field. Through repetitive deposition and processing of material layers using computer-controlled equipment, it is possible to produce complex, reproducible structures with predefined pore size and porosity. Moreover, 3D bioprinting—the creation of cell-laden 3D objects with controlled cell positioning while maintaining cell viability and functionality—introduced another level of sophistication. These bioprinting techniques use bioinks, which are mixtures of cells and biomaterials (mainly hydrogels) that can be printed into 3D cell-laden constructs (Schwab et al. [10], Aimar et al. [11]). Current bioinks are not always able to resemble the designed shape during the printing process or post-processing steps due to shear-thinning or other rearrangements in the microstructure of the hydrogel (Klar et al. [12], Temirel et al. [13]).

Shape fidelity is important, not only to fit the patient's defect for proper implantation accuracy, but also for cell behaviour. Cell behaviour can be affected by the three-dimensional environment, in which pore and strut sizes play an important role, as well as pore shape (Bidan et al. [9], Sobral et al. [14], Kačarević et al. [15]). In craniofacial regeneration, where restoration of bone and soft tissue structures demands exceptional anatomical accuracy, reproducible macrotopographic characterisation becomes particularly useful.

Recent advances demonstrate the potential of personalised composite scaffolds to accelerate craniofacial bone formation without added growth factors (Kim et al. [16]), and reviews highlight the utility of hierarchical and biomimetic designs for craniofacial applications (Gu et al. [17], Liu et al. [18]). This makes reproducible characterisation potentially valuable where scaffold geometry must replicate complex anatomical features to support clinical outcomes, like effective osseointegration (Lee et al. [19]). It also highlights a key consideration in the development of hydrogels (Habib et al. [20]), particularly for craniofacial use, where maintaining shape fidelity directly influences structural integration and tissue regeneration (Trubelja et al. [21]).

Generally, the shape fidelity of a material is assessed quantitatively using scanning electron microscopy (SEM, Olevsky et al. [22]), mechanical tests, and cell adhesion and proliferation assays (Scocozza et al. [23], Bornschlegl and Dietz [24]), including quantitative image analysis of the fabricated scaffold to compare the design's size and shape to the final product (Habib et al. [20], Habib and Khoda [25], Rossberg et al. [26]). Image analysis and pore measurements can be performed manually or semi-automatically using widely adopted software such as ImageJ (National Institutes of Health, Maryland, USA) or CellProfiler (Broad Institute, Cambridge, USA). However, these results often carry human error, also referred to as user bias, which can compromise data validation (De Boodt et al. [27], Murphy et al. [28]). This misinterpretation and lack of stable data processing are subject to systematic patterns of deviation that can lead to incorrect inferences and inaccurate research data.

By reducing measurement error and operator-dependent variability, the effects of different architectures on tissue regeneration can be studied more efficiently. This will enable the development of better bioinks and scaffolds tailored for clinical translation. Hereby, we propose an integrated hardware-software framework to assist researchers in acquiring high-quality (HQ) images of scaffolds and automating the analysis of features such as scaffold size, pore areas, and perimeters, reducing the operator-dependent variability associated with conventional manual measurement.

Unlike conventional image-based systems that rely on external, uncalibrated ambient lighting and generic software, the novelty of this framework lies in its mathematically constrained, integrated hardware-software loop. By coupling a dedicated 3D-printed illumination geometry with a custom shape-penalisation algorithm, the platform standardises the pixel-to-physical boundary transition, offering a highly reproducible engineering solution accessible to standard laboratories.

## Materials and methods

### Hardware setup

**Fig. 1:**
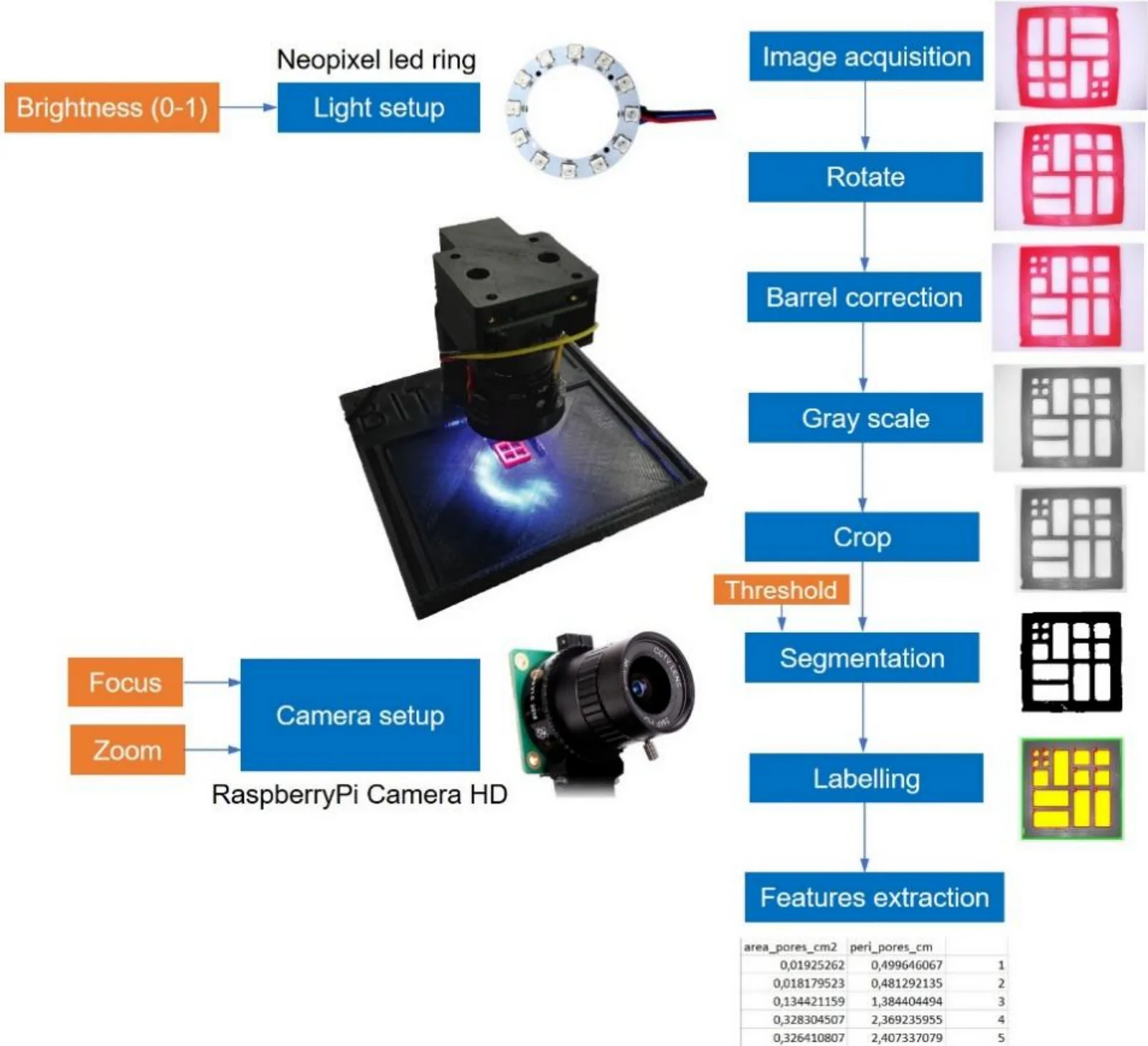
Schematic workflow of algorithm depicting each working block that comprises it. The images next to the block represent the outcome upon the step. Inputted variables are represented by orange blocks. The final result are labelled images with the measurements on the spreadsheet (csv file). The overall configuration and working setup is presented in this image, portraying the Raspberry HQ camera actively acquiring images of the scaffold (red) to be then processed by the software. We begin by calibrating the system’s illumination, focus and zoom to optimally acquire high pixel resolution images then fed to the algorithm to correctly extract the features (perimeter, area, and compactness)

The newly designed framework comprises three primary parts: a camera mount, a ring-point lighting system, and dedicated scaffold analysis software see Fig. 1). To maintain high resolution, we incorporated a 12.3 MP Raspberry Pi High Quality Camera (Sony IMX477 sensor) fitted with a Raspberry Pi 6 mm wide-angle CS-mount lens. A Raspberry Pi 3 Model B + (Raspberry Pi Foundation, Cambridge, UK) and an Arduino Uno (Arduino SRL, Monza, Italy) were utilised to establish the connection between the software and hardware components. The connection between the camera and the Raspberry Pi board allowed the system to read frames that were projected onto a computer screen as continuous visual images of each sample.

A NeoPixel 12-LED RGB ring (Adafruit Industries, New York, USA) with a custom 3D-printed adapter was incorporated to compensate for insufficient brightness and shadows that could compromise sample processing. Additionally, the LED ring provides homogeneous illumination against the high-contrast black-and-white background, increasing contour sharpness along the scaffold edges. By adjusting the light intensity levels, the camera was able to capture high-contrast images characterizing the entire area of the scaffold. The 37 mm diameter LED ring uses a single microcontroller input pin to control the entire chain. Power is supplied via a 5 V connection from the Arduino Uno board. Brightness is adjusted to a desired normalised value, ranging from low (logic '0') to high (logic '1') intensity.

The camera mount stabilizes the device during image acquisition. To design the support structure, factors such as dimensions, fittings, and acting forces were considered using Computer-Aided Design (CAD) software. The model stabilizes the camera while providing adjustable height settings to accommodate a range of scaffold sizes within the field of view. The LED ring adapter fits both the ring and the lens of the camera to provide direct illumination to the regions of interest (ROIs). The support system was printed using poly-lactic acid (PLA) for the monopod and base, and thermoplastic polyurethane (TPU) for the flexible LED ring support. PLA was used to print the monopod and the base. TPU was used for the led ring support provided of the good flexibility properties. Total printing time and filament length used varied for each part (refer to the supplementary data to access STL files).

### Software architecture

A custom MATLAB interface was developed, comprising three distinct scripts designed to perform image calibration, acquisition, and analysis. Once the hardware connection was established, the first step involved regulating the brightness of the LED ring via the Arduino board controlling the RGB values. Following this, the real-time pre-visualization of the camera feed was initiated to assess frame quality. While running the live video stream, the lenses were manually adjusted to optimize focus, depth of field, and aperture. Once calibration was complete, the system was ready for automated acquisition and processing.

### Image processing pipeline

The pipeline described below operates on a single zenithal image and therefore quantifies the two-dimensional projected macrotopography of the uppermost printed layer; internal three-dimensional architecture and pore interconnectivity are not resolved.

First of all, the hardware configuration was addressed, involving the adjustment of both the light and camera setups. The brightness of the LED ring was regulated by inputting a normalised value. The optimal brightness intensity was found to be k = 0.05 (as predetermined by the NeoPixel documentation), utilising a white colour across all 12 LEDs. A script was run to secure the interface and connection between the camera and the board. Once connected, the lens could be manually adjusted to achieve the desired focus; this was followed by the execution of another script that enabled a live stream of frames. The software then relied on a single inputted parameter: the black-and-white threshold (Otsu's method).

1. *Image acquisition*. The system takes a single snapshot frame using the HQ camera with a standardised pixel resolution. Through pixel formatting, the image was captured with a standardized resolution and displayed in the computer.
2. *Image rotation*. Due to the inverted positioning of the camera relative to the mounting board, the algorithm rotates the captured image 180° counter-clockwise using nearest-neighbor interpolation to modify the spatial pixel arrangement.
3. *Barrel effect correction*. In this last step, the algorithm corrected all distortion and warping effects. The zoom lens introduced a barrel distortion due to distance magnification, which was reduced via lens distortion correction routines (Ojanen [29], Vassy and Perlaki [30]). This caused the image to appear mapped around a cylinder or sphere. Radially symmetric distortions due to the barrel effect were corrected using the following distortion model:
  1 $$\widehat{r}-f\left(r\right)=r\times \left(1+k\times {r}^{2}\right)$$
  where:

*r̂*: is the distorted distance from the center of the image.

*r*: is the undistorted distance from the center of the image.

*k*: is the distortion parameter.

The distortion was removed by applying the inverse transformation of the model, expressed as:

2 $$r={f}^{-1}\left(\widehat{r}\right)$$

The distortion parameter, *k*, governs the overall behaviour of the distortion. A value of *k* = −0.15 was chosen from empirical testing; this value is specific to the 6 mm lens and working distance used here, and must be re-determined for a different optical configuration. The algorithm corrected these geometric errors and fitted the interpolation beyond the array edges to resample the radial symmetry of the image.

4. *Grayscale conversion.* The color image is converted to grayscale using the standard weighted transformation:
  3 $$IG=0.2989\times R+0.5870\times G+0.1140\times B$$
  Subsequently, the contrast between pixel intensities was increased to better distinguish the dark and light regions, thereby highlighting the scaffold layers against the background. Overall, the image was converted into a single-colour logical matrix, mapping pixels from white (1) to black (0).
5. *Image cropping.* Regions outside the scaffold perimeter are removed to improve tracking efficiency. The user can interactively select a Specifying Crop Region (SCR) position vector via a bounding cursor. The spatial coordinates define the starting position, width, and height of the cropping window.
6. *Image segmentation.* Image segmentation is crucial when pre-processing images to define the ROI. To proceed with the segmentation, a greyscale image must be input so the algorithm is able to differentiate between the background and the ROIs. Prior to image segmentation, a ground-truth image or training example is established as a reference to determine what is considered background. Choosing black or white PLA to print the support ensures high contrast between the sample and the background. For this reason, the algorithm also performs a color inversion (complementation) depending on the colour used. This enables the system to clearly differentiate the sample pores from our ROI (the scaffold and its pores). Using Otsu's global thresholding method, pixels are binarized into 1 (ROI/pores) or 0 (background) based on maximizing the inter-class variance in the image histogram
  4 $$Thr=\mathrm{m}\mathrm{a}\mathrm{x}({\sigma}_{b}^{2}(t))$$
  where the inter-class variance is defined as:
  5 $${\sigma}_{b}^{2}(t)={\omega}_{0}(t)\cdot {\omega}_{1}(t)\cdot {\left[{\mu}_{0}(t)-{\mu}_{1}(t)\right]}^{2}$$

Thus, the algorithm can be outlined as described below:

**Otsu’s Algorithm**

**Figure:**
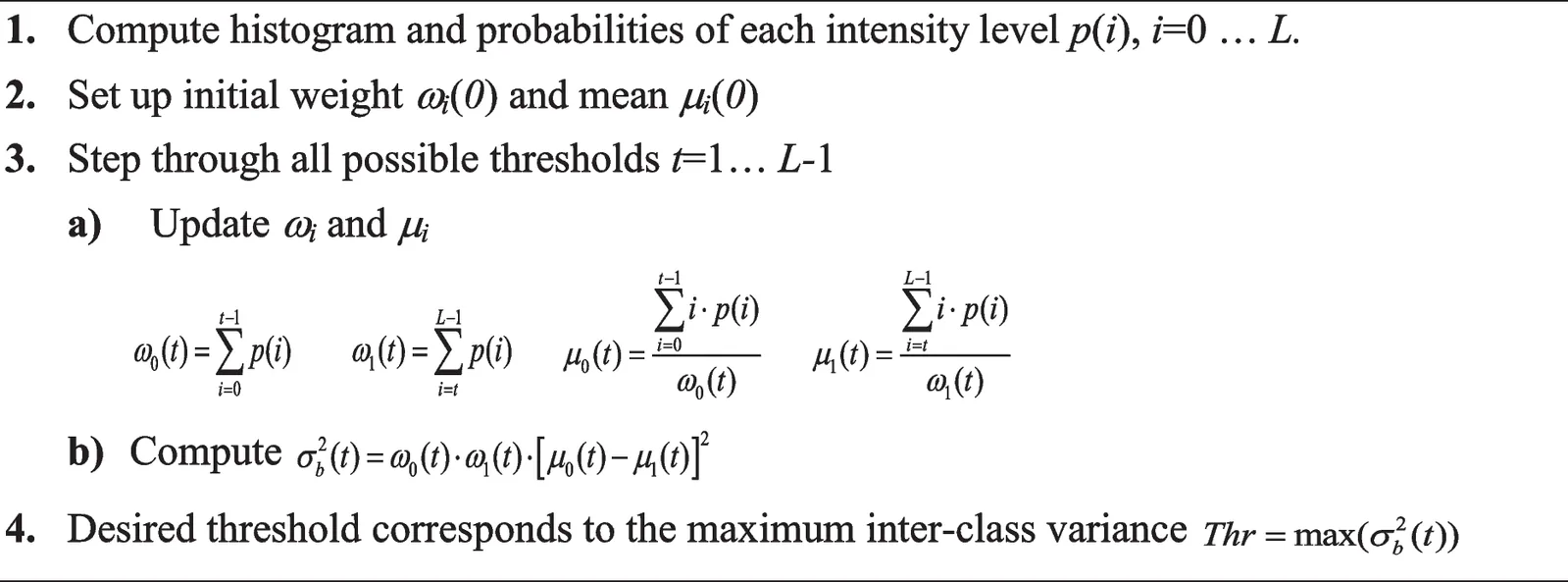

Once the operation was complete, a binarized image was obtained automatically (see Fig. 1). Nevertheless, the user interface enabled manual adjustment of the threshold using a slider.

7. *Labelling.* The labelling algorithm identifies independent components based on the connectivity of pixels adjacent in a horizontal, vertical, or diagonal sequence (Haralick and Shapiro [31]). It reads the single-value pixel intensity to identify regions with high contrast (scaffold-background). Afterwards, neighbouring components are analysed to locate common edges and identify each object. An 8-connectivity value is used to locate pixels that share edges and can be grouped into the same object. The output image is a label matrix of contiguous regions, with the same size as the binary input image (segmented image).
8. *Feature extraction & blob analysis.* Feature extraction is performed based on pore count and detection. Once the image has been segmented and labelled, the feature extraction step returns the number of pores and computes the area and perimeter of each in pixels. The system then generates an array of the pores that fulfil the condition as a Boolean true (logical ‘1’). Once all elements are targeted, they are classified as either pores or scaffolds according to their area. Afterwards, blob analysis is applied to remove elements that are too small or too large. Following the filtering of these connected components, two datasets are created for the area and perimeter; the values that meet the condition are subsequently converted to centimetres:
  6 $$x\hspace{0.33em}(c{m}^{2})={A}_{soft}(pi{x}^{2})\cdot \frac{1\hspace{0.33em}(c{m}^{2})}{{A}_{meas}(pi{x}^{2})}$$

It is important to note that *A*_*meas*_*(pix*^*2*^*)* is the number of *pixels* found in 1 cm^2^, scaled manually and adjusted depending on the height of the monopod. Accordingly, the variable *A*_*soft*_*(pix*^*2*^*)* is the measurement computed by the software in *pixels*^*2*^. The conversion from *pixels*^*2*^ to cm^2^ is performed by dividing the area obtained by the software (*A*_*soft*_) by the known pixel density per cm^2^ (*A*_*meas*_) using cross-multiplication. The algorithm highlights the entire identified pore area (in yellow), then adds eye-guided labels to the pores (in red) and the scaffold (in green). These bounding boxes fitting each element serve only as an indicator of pore or scaffold detection; they are not involved in the measurement. Ultimately, an overlay within the greyscale image is fitted to serve as a visual guide for the user (see Fig. 1).

Once processed, the data is stored in a comma-separated values spreadsheet (csv file) and saved alongside the processed image in the current directory path. The output image boxes present an assigned number indicating the location of those pores, which corresponds to the pore computations on the spreadsheet. This spreadsheet delivers a fully quantified data projection of the scaffold pores and their status to researchers. Object description characterises the dimensions of a polygon by extracting features or descriptors such as location, orientation, and size, with area and perimeter being the most frequently used. The calculated characteristics refer to the scaffold pores and include the area, perimeter, and compactness. Based on these two measurements, the compactness (*C*) of a region can be determined. This parameter is defined as a measure of shape, representing the efficiency within the boundaries enclosing the area, and is mathematically known as the isoperimetric quotient. Theoretically, a convoluted shape becomes more compact if region pixels move further away from the centre of gravity to fill gaps, or by adjusting its sides closer to the centre of gravity (Nixon and Aguado [32]). In addition, while the area does not discriminate if a defect is concentrated at a single point, compactness can determine it. Compactness is a measure of shape correlated solely to its radius:

7 $$C(s)=\frac{A(s)}{P{(s)}^{2}/4\cdot \pi }$$

By the isoperimetric inequality, Eq. 7 is bounded on (0, 1]: a circle is the most compact shape and gives C(circle) = 1, whereas a perfect square gives *C*(square) = π/4 = 0.7854. Because the target geometry of the printed pores is a square rather than a circle, the quotient is rescaled so that a perfect square, and not a circle, is anchored at unity. This gives the square-normalised compactness used throughout this work:

8 $${C}_{e}(s)=\frac{C(s)}{\pi /4}=\frac{16\cdot A(s)}{P{(s)}^{2}}$$

where *Cₑ* is written C_custom in the text and figures. Accordingly *Cₑ*(square) = 1 and *Cₑ(*circle) = 4/π = 1.2732, so that Cₑ is bounded on (0, 4/π]. We note that Cₑ is the reciprocal of the printability index *Pr* = *P*^2^/(16A) commonly used to assess bioink shape fidelity (Habib et al. [20]), which allows the values reported here to be related to that literature. Finally, departure from the square target in either direction—a pore whose boundary is rounder than a square, or one that is more convoluted—is penalised symmetrically and expressed as a percentage quality score, *Cₚ(k*):

9 $$\left\{\begin{array}{lc}{C}_{p}(k)=\left|\frac{400\cdot \left|C(k)\right|}{\pi }-200\right|& \forall C(k)\ge \pi /4\\ {C}_{p}(k)=\frac{400\cdot C(k)}{\pi }& \forall C(k)<\pi /4\end{array}\right.$$

A perfect square pore therefore yields *Cₚ* = 100%, a perfectly circular pore yields *Cₚ* = 72.7%, and *Cₚ*(*k*) is bounded on [0, 100]. Expected values for reference geometries are given in Table 1.

**Table 1:** Expected values of the compactness metric for reference geometries. *C* is the isoperimetric quotient (see Eq. 7), *Cₑ* its square-normalised form (see Eq. 8) and *Cₚ* the penalised percentage quality score (see Eq. 9)

Pore shape	C (Eq. 7)	Cₑ (Eq. 8)	Cₚ (Eq. 9), %
Perfect square (target geometry)	0.7854 (π/4)	1.0000	100.0
Regular hexagon	0.9069	1.1547	84.5
Circle	1.0000	1.2732 (4/π)	72.7
Equilateral triangle	0.6046	0.7698	77.0
Highly convoluted boundary	→ 0	→ 0	→ 0

Moreover, categorisation in a colour-indexed graph displays colour-gradient pores according to the projected compactness. Subsequently, the average is weighted to obtain an overall indicator of the scaffold pore condition. These aspects enable researchers to quantitatively characterise and classify pores based on their shape.

In summary, the image-processing pipeline proceeds from objective segmentation to geometry-specific feature extraction. Otsu’s method performs an automated global sweep that maximises the inter-class variance (σ^2^B) and therefore determines a threshold independently for each image, which reduced the influence of illumination and material reflectivity across the substrates tested here (PLA, silica–gelatin hydrogels and silica). The binarised matrices are then processed through the square-normalised, geometry-penalised compactness metric of Eqs. 8 and 9, which anchors a perfect orthogonal pore configuration at unity and enables bi-directional quantification of morphological anomalies such as circular warping, wall erosion or directional print shearing, at a lower processing and licensing overhead than general-purpose image analysis platforms.

### Cost and software requirements

A costed bill of materials for the imaging rig is given in Table 2. The total hardware cost is approximately €170 (US$190) excluding value added tax, based on list prices from official distributors at the time of writing; including 21% VAT the total is approximately €205. This figure excludes the fused-deposition-modelling 3D printer used to produce the mount, the host computer, and software licences. Consumable filament is costed on a per-gram basis.

**Table 2:** Bill of materials. Prices are list prices from official distributors retrieved in August 2026 and exclude VAT. The two columns are manufacturer list prices in each currency and are not currency conversions of one another

Component	Supplier	€ (ex VAT)	US$ (ex VAT)
Raspberry Pi 3 Model B + single-board computer	Raspberry Pi	34.37	35.00
Raspberry Pi High Quality Camera (12.3 MP, IMX477)	Raspberry Pi	43.61	50.00
Raspberry Pi 6 mm wide-angle lens, CS-mount	Raspberry Pi	21.64	25.00
NeoPixel Ring, 12 × 5050 RGB LED, 37 mm	Adafruit	7.79	8.95
Arduino Uno Rev3	Arduino	24.02	27.60
microSD card, 32 GB, class 10/A1	various	16.00	18.00
5.1 V/2.5 A micro-USB power supply	Raspberry Pi	5.80	8.00
PLA filament (≈150 g: monopod and base)	consumable	3.27	3.75
TPU filament (≈20 g: LED-ring adapter)	consumable	0.56	0.64
Jumper wires, breadboard, M3 fasteners	various	13.00	15.00
Total		170	190

Image analysis was implemented in MATLAB R2024b (MathWorks, Natick, MA, USA) and requires the Image Processing Toolbox; both are commercial products. Communication with the Raspberry Pi and Arduino boards uses the MATLAB Support Package for Raspberry Pi Hardware and the MATLAB Support Package for Arduino Hardware, which are available at no additional charge to licensed MATLAB users through the MATLAB Add-On Explorer. The Computer Vision Toolbox is not required, because the radial distortion correction is implemented directly from Eqs. 1–2 rather than through a camera-calibration object. Because the pipeline uses only standard image-processing primitives—Otsu thresholding, connected-component labelling and region property extraction—it can also be reproduced without a commercial licence using GNU Octave with the image package, or Python with OpenCV/scikit-image.

## Discussion and results

The performance of the platform was assessed in four assays. First, a printed reference grid with a known square side length of 0.5 cm was imaged to quantify the trueness and the repeatability of the segmentation and measurement chain; low dispersion among the measured pore areas indicates high repeatability, while the offset from the nominal area quantifies trueness. Second, a 3D-printed PLA scaffold containing three classes of pores of different size and shape was used to evaluate pore detection and repeatability across *N* = 5 acquisitions; the same specimen was then measured manually by three operators experienced in tissue engineering (TE) under standard conditions, so that intra-observer (within-user) and inter-observer (between-user) variability could be compared with that of the platform. Third, two silica–gelatin hybrid bioink scaffolds were imaged to examine performance on hydrated constructs with non-linear pore boundaries. Fourth, a silica-based scaffold was used to demonstrate the compactness output. The third and fourth assays were performed without an independent reference measurement of pore geometry and are therefore reported as feasibility demonstrations rather than accuracy validations.

As previously explained, shape fidelity can be used to evaluate the printability of an ink or hydrogel, and thus the resolution of the cross-linking density. Consequently, these metrics can indicate biological performance across different concentrations, which is crucial for bioink development. In this context, shape fidelity describes the overall filament spreading versus retention upon extrusion compared to the CAD design, a concept sometimes referred to as print accuracy. By providing compactness values, the software serves as a predictor of shape fidelity, allowing researchers to evaluate both printer accuracy and scaffold condition. As a primary indicator of scaffold stability and gelation quality, assessing these parameters enables researchers to monitor ink quality, thereby optimising the biofabrication process and accelerating their research.

### Methodological context and alternative modalities

Traditional high-fidelity characterisation techniques, most notably micro-computed tomography (micro-CT), offer exceptional three-dimensional internal architectural mapping. However, micro-CT is constrained by high instrument and operating cost and by limited accessibility (Bertoldi et al. [33], Martinez-Garcia et al. [34]); by long acquisition and reconstruction times, reaching 19.5 h and 166 GB of data per specimen at the finest pixel sizes (Cengiz et al. [35]); and, for hydrated hydrogels, by the dehydration, shrinkage and contrast-staining artefacts associated with imaging in the wet state (Kwon et al. [36], Martinez-Garcia et al. [34]). Image-processing workflows for CT of polymeric scaffolds with cells continue to be developed (Williams et al. [37]). The optical imaging platform presented herein is not intended to replace volumetric 3D analysis, but to complement it by providing a rapid, non-destructive and accessible screening tool. It should be emphasised that the two modalities measure different quantities: micro-CT resolves internal three-dimensional architecture, pore interconnectivity and through-thickness geometry, whereas the present platform quantifies the two-dimensional projected macrotopography of the uppermost printed layer from a single zenithal view. Feasibility was examined across mechanically distinct substrates, including rigid polymers (PLA), hydrated silica–gelatin hybrids and brittle silica-based scaffolds.

### Gridded paper assay

**Fig. 2:**
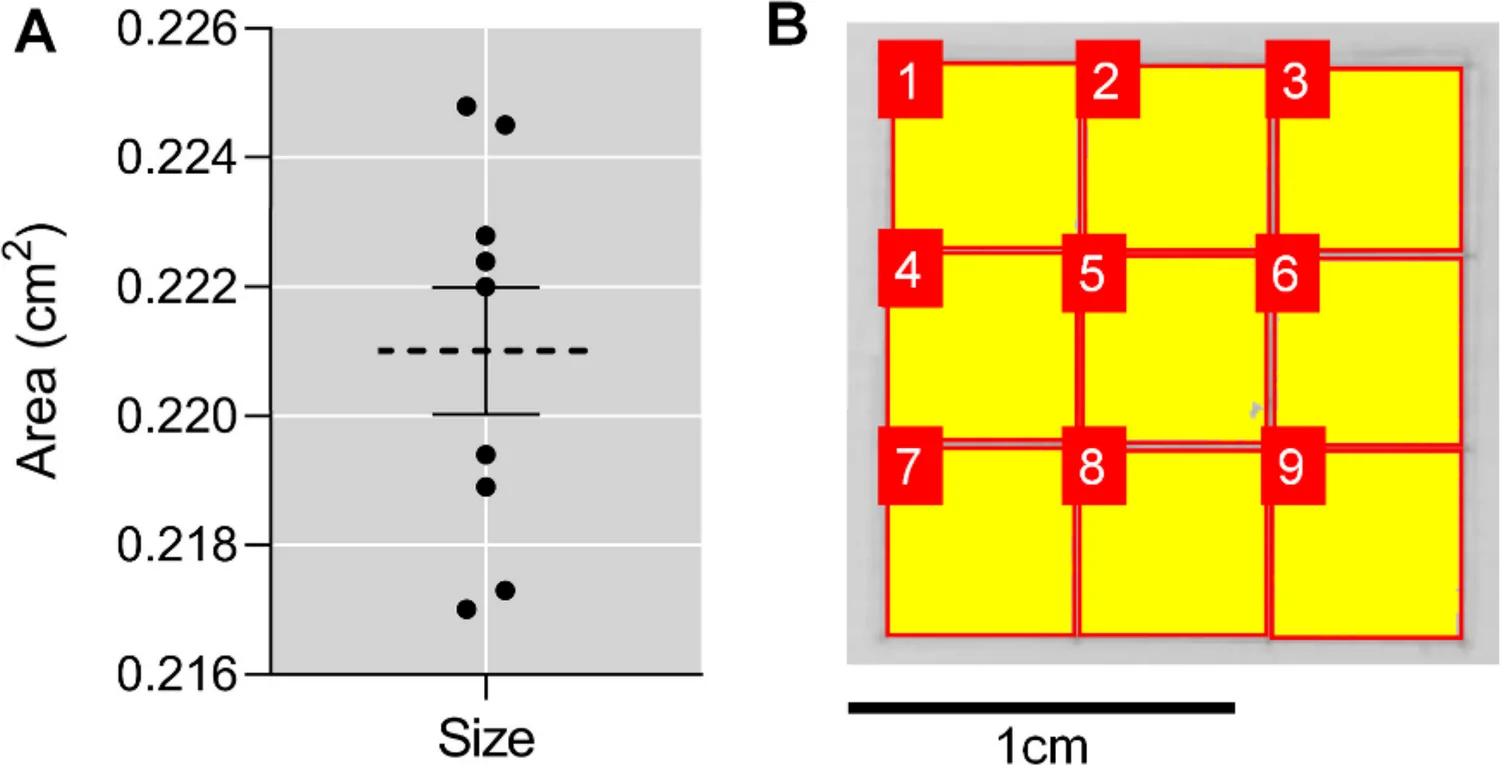
Software fidelity assay on 0.5 cm gridded paper. **A**) Area deviation among pores. *σ* = 0.002948 cm^2^*, μ* = 0.221 cm^2^, *CV* = 1.33%*.* **B**) Area and pores detected by the computer vision system on the gridded paper

The primary goal of the tool is to demonstrate a low degree of deviation; the first assay (see Fig. 2), utilising the gridded paper, helps validate this parameter. The software acquired and processed a 5 × 5 mm section of gridded paper, depicted on the right-hand image in Fig. 2. The adjacent chart portrays a minor or relatively low deviation from the theoretical value, yet exhibits high fidelity due to a low coefficient of variation (*CV* = 1.33%).

Using the known areas of the grid, trueness and repeatability can be assessed separately. The mean measured pore area was μ = 0.221 cm^2^ against a nominal 0.250 cm^2^, that is, an absolute error of 0.029 cm^2^ and a relative error of − 11.6%: the platform systematically underestimates pore area. Repeatability, by contrast, was high (SD = 0.0029 cm^2^; CV = 1.33%). The bias is attributable to the manual pixel-per-centimetre calibration and to boundary assignment at the segmentation threshold, which places the detected edge inside the true pore edge; because it is systematic and reproducible it can be removed by calibrating the system against a reference of certified area. Each pixel accounts for a specific fraction of the total area, which is a relevant consideration when quantifying small pores.

### PLA-based scaffold assessment

**Fig. 3:**
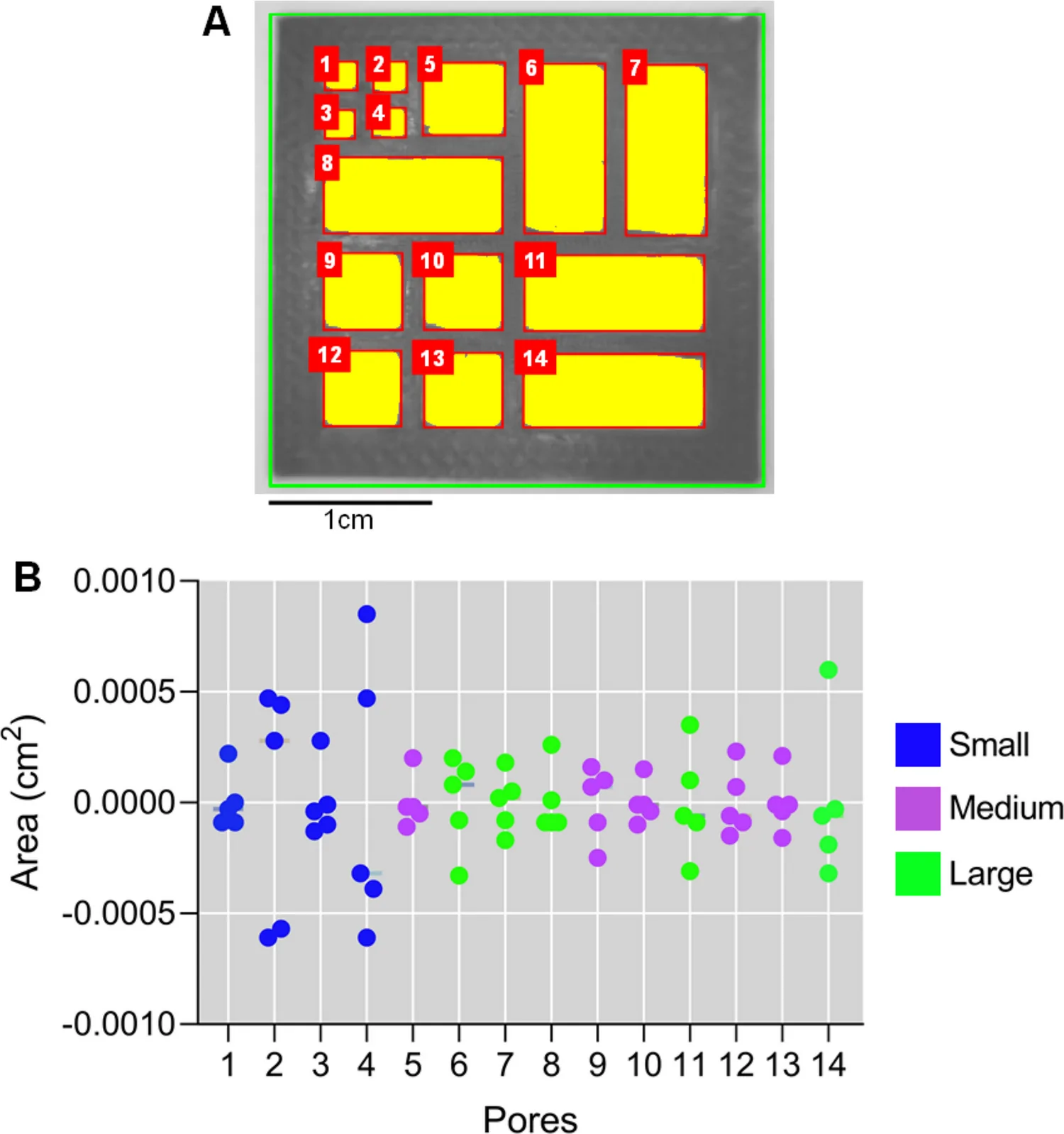
**A**) Pores detected by the computer vision system on 3D printed scaffold. **B**) Area fidelity test on 3D printed scaffold. Area calculated for each pore from 5 trials. Pores with relatively higher variance are those with a smaller size. *CV* = 0.01*%*−2.48*%*

Standardisation and homogeneity were evaluated to determine whether the system deviates or maintains consistency when trialed multiple times (*N* = 5) on the same scaffold. The second assay enabled the evaluation of this consistency, with a low coefficient of variation (*CV* = 0.01%—2.48%). Figure 3A shows the porosity detected by the device, with the disparity being less than one-tenth of a millimetre (Fig. 3B). There appears to be greater discrepancy among the smaller pores. As expected, the fewer pixels a pore covers, the higher the relative weight each pixel accounts for. Consequently, the conversion from pixels to centimetres is greatly affected by this adjustment when running the script repeatedly.

Measurement error is conditioned by the deviation from the ground truth. In these types of experiments, the ground truth is either known or projected. Projections are established from a series of evaluations and agreed upon through statistical analysis. Furthermore, we considered both intra-observer (within-user) and inter-observer (between-users) variability. This subjectivity is linked to an individual's ability to scrutinise each pore, making experience a critical factor. Hence, conventional techniques were tested on the same assay to benchmark the true potential of our method.

**Fig. 4:**
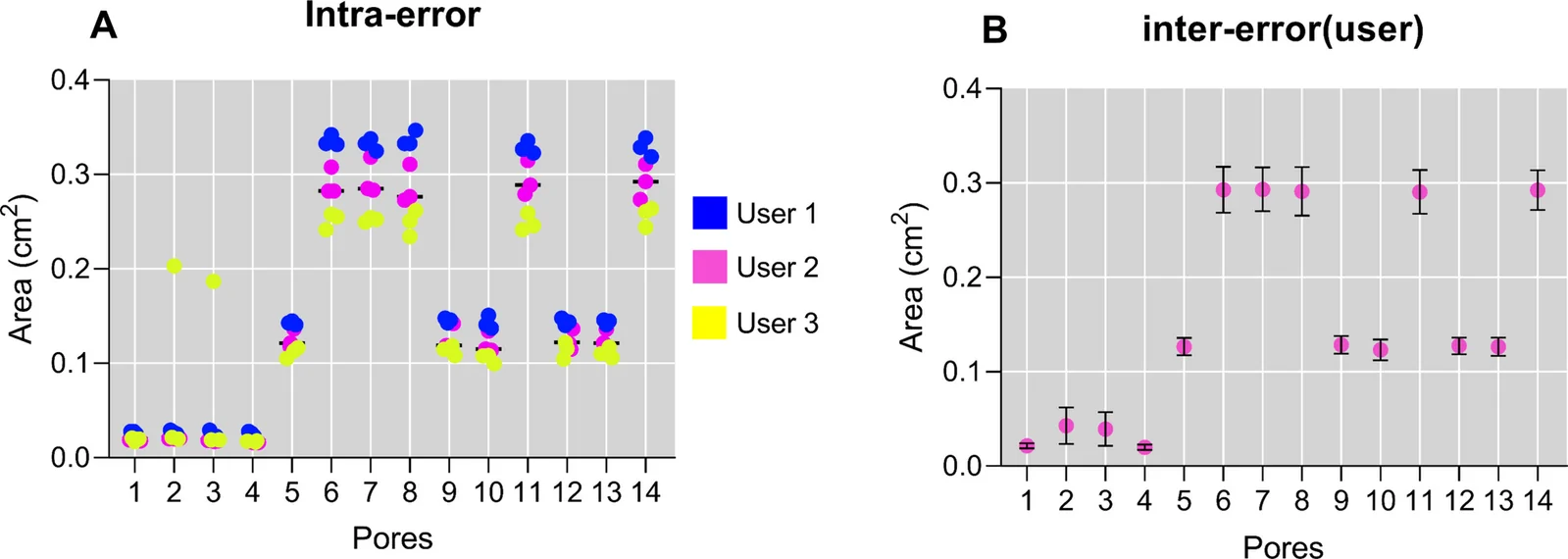
User fidelity test on 3D printed scaffold. **A**) portrays dispersion, conveyed intra-relationally, over the *y*-axis (area) for each user in 3 trials performed. **B**) portrays dispersion, inter-relationally, again over the *y*-axis on the average for the 3 users *CV* = 11.51%−22.76% for the inter-error, and a *CV* = 0%- 12.68% for the intra-error

The results in Fig. 4A show an interesting trend: rather than portraying high discrepancy among smaller pores, the manual measurements did so on the larger ones. This low degree of agreement is further confirmed by the inter-error (see Fig. 4B). A coefficient of variation of *CV* = 11.51%−22.76% for the inter-error, and a *CV* = 0%−12.68% for the intra-error, evidence the higher divergence we aimed to reveal in this assay. This divergence exposes a systemic problem in collaborative laboratory environments—a tiresome approach that requires full attention and experience, which, if not fully accounted for, leads to user bias. The perceived trend is concentrated on the larger pores, which show higher dispersion than the others, as corroborated by the error bars in the graph (see Fig. 4B).

The dispersion of the manual measurements is substantial within these limits, with changes exceeding an order of magnitude. These observations are consistent with our hypothesis that human-assisted measurement is subject to variability that can lead to mismatches or data loss. Under the conditions tested, the repeatability of the platform did not display the pore-size dependence seen in the manual measurements; this was assessed on a single specimen and should not be generalised.

### Biomaterial-based scaffold

**Fig. 5:**
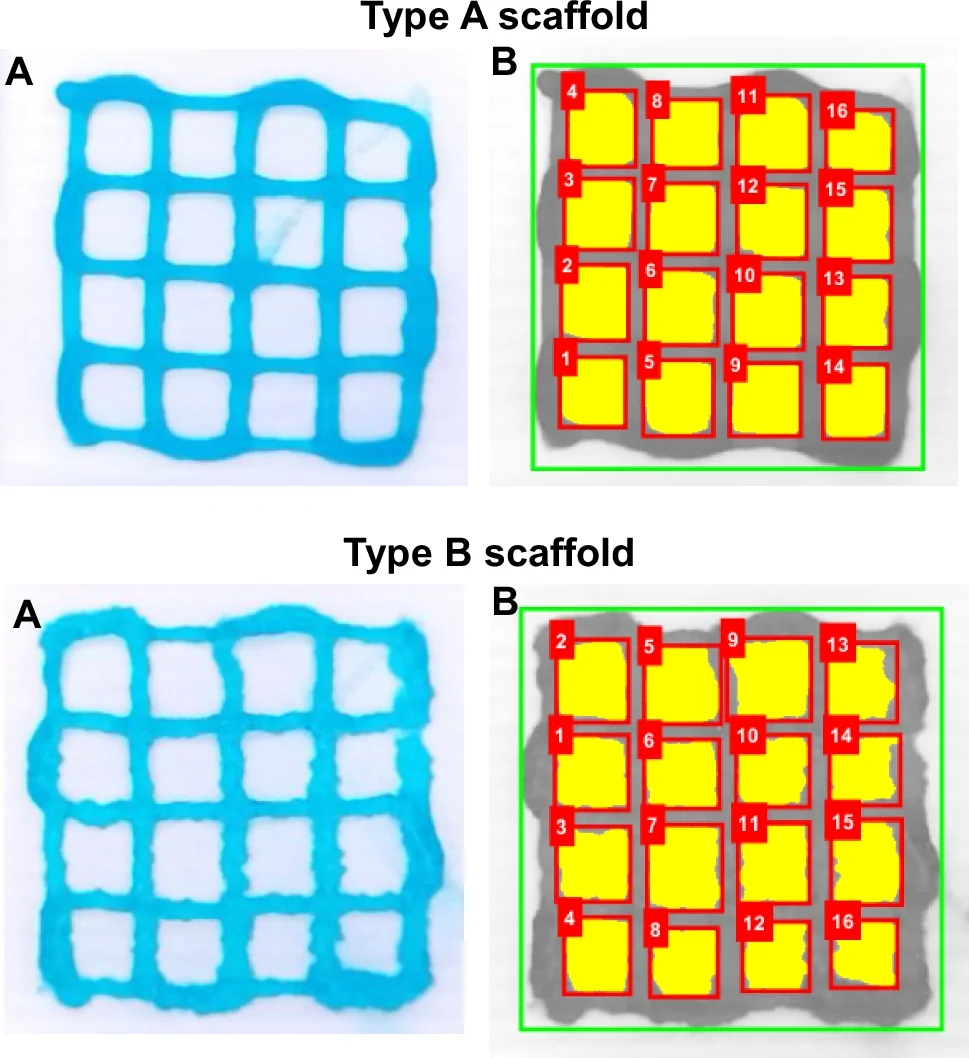
Silica–gelatin hybrid bioink scaffolds of two different compositions tested with the custom software: Type A (gelatin:sol 1:1) and Type B (gelatin:sol 0.5:1). For each ink, **A**) photographic image of the printed scaffold and **B**) the porosity detected by the computer vision system

Subsequently, we assessed the software under actual laboratory settings using biomaterial-based scaffolds with geometrically complex pores. Two different bioinks (Type A and Type B) were utilised to print a scaffold in order to analyse the pore number and size using the developed software (see Fig. 5). The inks were dyed blue using methylene blue at a concentration of 50 μL per 5 mL of ink. This was performed to create contrast between the printed scaffold and the surface, as the inks are originally transparent. A 15 × 15x3 mm scaffold was 3D-printed using a Cellink BioX printer (BICO Group, Boston, USA) at a speed of 6 mm/s and an extrusion rate of 2.50 μL/s through a 0.58 mm nozzle. The infill density of the scaffolds was set at 20%, yielding a 16-pore structure. Once printed, a photograph was acquired, and the software analysed the number and size of the pores.

Both bioinks were self-setting silica–gelatin hybrids prepared by the sol–gel route and developed and characterised in detail elsewhere (Rodríguez-González et al. [38]). Sols were prepared from tetraethyl orthosilicate (TEOS) and (3-glycidyloxypropyl)trimethoxysilane (GPTMS) at a TEOS:GPTMS volume ratio of 8:2, with 0.1 M HCl and distilled water at a water/TEOS molar ratio of 8, stirred at 700 rpm until homogeneous. Type A gelatin (from porcine skin) was dissolved in water at 20% w/v at 35 °C and adjusted to pH 4.5 with 1 M HCl to control the gelation rate. Sol and gelatin were combined at 35 °C and stirred at 500 rpm until the onset of gelation. No external cross-linking agent, photoinitiator or ionic bath was used: the inks are self-setting, with GPTMS acting as a covalent coupling agent between the silica network and the gelatin through epoxide ring-opening, and the setting rate controlled by pH and temperature. The two inks differ only in the gelatin:sol volume ratio, 1:1 for Type A and 0.5:1 for Type B, corresponding respectively to the compositions designated T8-G1 and T8-G0.5 in ref. [38]. After printing, the scaffolds were kept in a hermetic box and matured for 7 days at room temperature to complete the sol–gel reaction.

Figure 5 shows the photographic images (A and B) and the porosity detected by the computer vision system for the Type A and Type B scaffolds, respectively. The results reveal the effect that composition has on pore geometry; the complex geometries of the hydrogel-based scaffolds differ significantly from the PLA-based design previously tested. Linear patterns are easier to quantify in contrast to these complex geometries with non-linear boundaries, where manual measurements are more prone to error. Herein lies the potential of using computer-aided image-processing software. Upon closer inspection of the curvatures and edges of both scaffolds, our software is able to identify the entire region (marked in yellow).

**Fig. 6:**
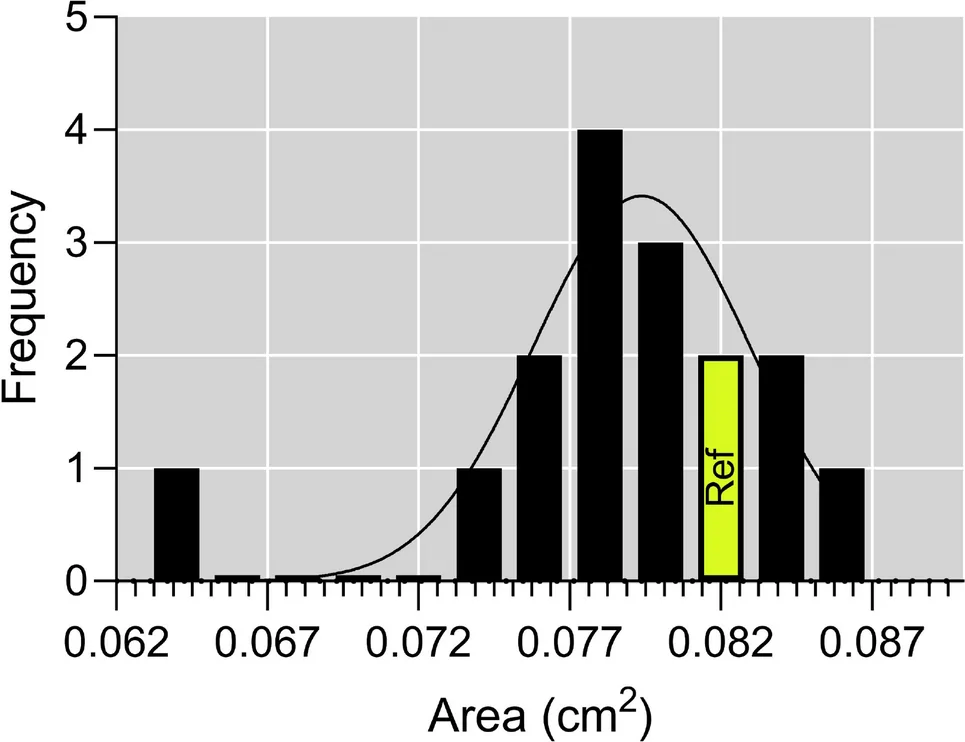
Histogram of the pore area deviation from hypothetical design ground truth, ref bar

Figure 6, shows the histogram of the pore areas (*N* = 16 pores), comparing the frequencies of values among the pores. It reveals a certain deviation from the inputted approximate strut width of 0.7 mm on the scaffolds (*N* ~ *σ* = 0.051 cm^2^ and *μ* = 0.0786 cm^2^) which is theoretically supposed to produce 0.0827 cm^2^ pores (design ground truth). By these means, the software reveals a printing resolution error of e = 0.0051 cm^2^, regarded as the standard deviation of the areas.

Depending on the interactions within the matrix, the scaffold's behaviour will differ, meaning its properties will vary accordingly. The molecular frameworks that define these interactions reflect the stability and degradability of the substrate. These screenings can provide the visual means to understand changes in topographical morphisms through time-dependent degradation or pore formation. Ultimately, by fully quantifying these transformations, it becomes feasible to better characterise bioinks and their properties prior to implantation.

### Scaffold compactness assay

**Fig. 7:**
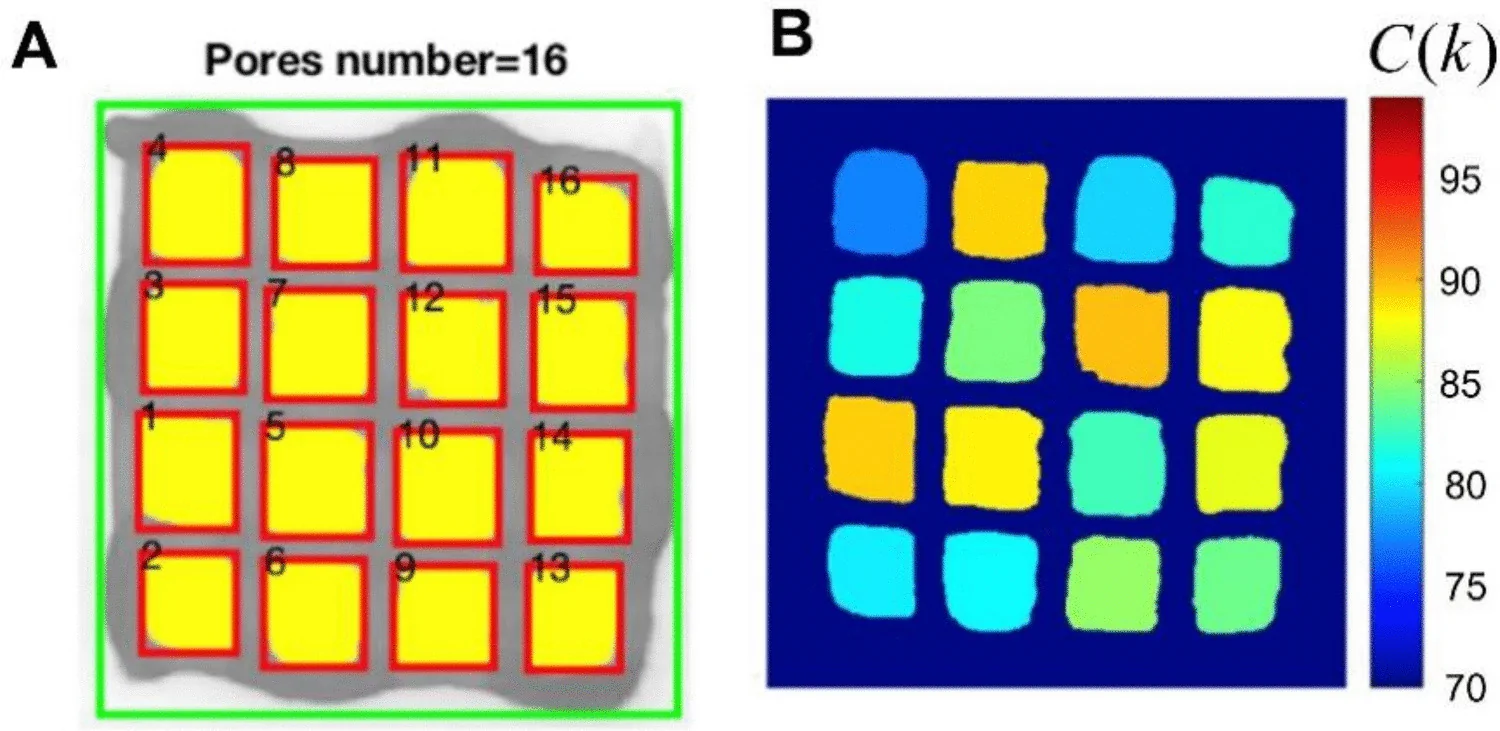
Compactness assay on a silica-based 3D-printed scaffold. **A**) Pores detected by the computer vision system, with the compactness percentage *Cₚ*(*k*) displayed within the bounding box of each pore. **B**) The same values rendered as discrete colour levels; the colour bar spans *Cₚ*(*k*), where 100% corresponds to a perfect square pore

Finally, we examined the compactness output on a silica-based scaffold, which on printing is expected to form pores of uniform and symmetric shape. Although the printer is set to maintain these constraints, within the scaffold matrix there is a clear deviation in the shape of the boundaries that form the pores (see Fig. 7). As no independent reference measurement of pore shape was available for this specimen, this assay is reported as a feasibility demonstration of the compactness output rather than as a validation of its accuracy.

In Fig. 7A, the numerical value of the compactness is displayed within the bounding box of the pore, while Fig. 7B displays this value as a discrete colour level. As previously explained, this references the value on a more visual colour index scale.

### Processing time

Processing time was not measured in this study, and no quantitative claim is made regarding it. The advantage demonstrated here lies in reduced user-dependent variability and in the number of pores quantified per specimen rather than in speed — which is also what the comparable literature actually supports: reports of efficiency in semi-automated pore-analysis tools are generally qualitative (Rossberg et al. [26]), and the one study that timed the complete operator workflow found no wall-clock advantage over manual ImageJ measurement, while reporting far more pores analysed and lower inter-user variability (Olevsky et al. [22]).

### Limitations

The present workflow is semi-automated rather than fully automated. Focus and aperture of the lens, the monopod height, the illumination level and the crop region are set by the operator; the Otsu threshold may optionally be adjusted through the interface slider; the pixel-per-centimetre scale is entered manually for each monopod height; and the radial distortion coefficient (*k* = − 0.15) was determined empirically for the 6 mm lens and working distance used here and must be re-determined for a different optical configuration. The platform therefore reduces, but does not eliminate, user-dependent variability, and the reduction reported here applies to the substrates and imaging conditions tested. The measurement is further restricted to the two-dimensional projected geometry of the uppermost printed layer: internal architecture, pore interconnectivity and through-thickness geometry are not resolved, and specimens whose contrast against the background is low may require staining, as was done here for the transparent hybrid inks. Finally, the trueness of the area measurement is limited by the manual scale calibration, which produced a systematic underestimation of approximately 12% in the reference-grid assay.

## Conclusions

This work presents a low-cost, semi-automated method for the quantification of scaffold surface macrotopography in a laboratory setting. Results from the four assays illustrate both the advantages of the tool and the limitations of the conventional manual approach. On the reference grid the platform was highly repeatable (CV = 1.33%) with a systematic and correctable underestimation of 11.6%; on the PLA scaffold, repeated acquisitions agreed to within CV = 0.01–2.48%, against an inter-user CV of 11.5–22.8% for manual measurement of the same specimen. Repeatability was therefore markedly higher than that of manual counting, which is the principal benchmark for the algorithm. Image quality remains a critical factor, as the variability associated with mobile phones or other camera devices of differing specification can degrade both the data and the visual output.

The instrument produces reproducible measurements and publication-quality graphics that facilitate the interpretation of the data, and it can filter out unwanted objects using a user-controlled range of minimum and maximum areas, with quality inspection at every step so that the user can validate the output. The measured per-specimen processing times are reported in Section 3.6.

A lack of experience in pore measurement often leads to less accurate approximations. The platform delivers high-quality images suitable for publication alongside measurements whose repeatability is independent of operator experience, which benefits both experienced and inexperienced researchers, particularly when analysing asymmetrical pores.

In conclusion, the proposed tool is a promising, accessible approach to the quantification of scaffold surface geometry. It can be applied to monitor changes in the regions that make up the scaffold over time, providing insight into behaviour prior to introducing the scaffold into a metabolic environment. Craniofacial tissue engineering, in which scaffold geometry must eventually replicate complex anatomical features, is a natural prospective application of such quality control; we emphasise, however, that no craniofacial-specific, patient-specific or clinical specimen was evaluated in this study, and that establishing utility in that setting will require dedicated validation against a reference modality.

Ultimately, by combining custom-calibrated structural illumination with a geometry-penalised feature extraction pipeline, this framework contributes a reproducible, non-destructive and low-cost methodology to biomedical automation engineering, and indicates a pathway towards objective quality control systems in scaffold fabrication.
